# Remimazolam-based anesthesia in a patient with hypertrophic obstructive cardiomyopathy undergoing radical colorectal cancer surgery: A case report

**DOI:** 10.1097/MD.0000000000037199

**Published:** 2024-03-01

**Authors:** Yongchang Shi, Chengchao Zuo, Yiwen Zhang, Chao Zhou, Fengjiao Zhang, Xuelian Zhao

**Affiliations:** a Department of Anesthesia, the Fourth hospital of Hebei Medical University, Shijiazhuang, PR China.

**Keywords:** cerebral functional status index, general anesthesia, hypertrophic obstructive cardiomyopathy, intravenous, remimazolam

## Abstract

**Background:**

The goal of anesthesia in patients with hypertrophic obstructive cardiomyopathy (HOCM) is to reduce the risk of left ventricular outflow tract obstruction triggered by anesthetics. Remimazolam is a newly developed anesthetic that has been reported to have superior hemodynamic stability. There have been no reports on the completion of non-cardiac surgery with remimazolam in patients with HOCM.

**Methods:**

Here we report the case of a 49-year-old man diagnosed with hypertrophic obstructive cardiomyopathy who underwent resection of colon cancer with remimazolam and remifentanil anesthesia. A bolus 0.3 mg/kg remimazolam was administered for anesthesia induction, and then adjusted to 2 mg/kg/h to maintain anesthesia. Set the pain threshold index to 50 to auto-control the infusion speed of remifentanil.

**Results:**

No hypotension occurred during anesthesia, and norepinephrine was not administered. After conversion to open surgery, the patient’s blood pressure elevated and reduced with urapidil and esmolol.

**Conclusion:**

In this patient with HOCM, remimazolam and remifentanil provided adequate anesthesia for induction and maintenance to complete the right hemicolectomy.

## 1. Introduction

Hypertrophic obstructive cardiomyopathy (HOCM) is a genetic disease characterized by asymmetric hypertrophy of the left ventricular myocardium, some even leading to obstruction of the left ventricular outflow tract (LVOT). It is important to prevent LOVT obstruction during general anesthesia (GA) by preventing potential hemodynamic instability in patients with HOCM. However, anesthetics cause cardiovascular depression by reducing systemic vascular resistance and cardiac contractility in a dose-dependent manner. Remimazolam is an ultra-short-acting benzodiazepines that is rapidly metabolized into inactive metabolites by nonspecific esterases.^[1]^ Remimazolam has superior hemodynamic stability compared to other intravenous anesthetic agents.^[2]^ There are no reports on the safety of remimazolam-based anesthesia in patients with HOCM. Therefore, we report the case of a patient with HOCM who underwent radical resection of colon cancer using remimazolam and remifentanil. Written, informed consent was obtained from the patient for the publication of this case report.

## 2. Case report

A 49-year-old male patient (168 cm, 62 kg) underwent laparoscopic right hemicolectomy with a history of radical transverse colon cancer surgery and postoperative chemotherapy. The patient was diagnosed with HOCM during the preoperative evaluation, and the ECG showed ST-T changes. The patient was monitored for invasive arterial BP, cardiac output, cardiac index, and stroke volume variation (SVV) (Edwards Lifesciences, Vigileo, CA), electrocardiography and pulse oximetry. In addition, the cerebral functional status index (CFSi, HDX-I, Yifeihuatong Corp, Beijing, China) was used to monitor the depth of anesthesia (DoA). Prior to induction, 0.3 mg/kg remimazolam was injected, then adjust to continuous infusion at 2 mg/kg/h; Set the pain threshold index (PTi, range 0–100) to 50 to auto-control infusion speed of remifentanil with the closed-loop automatic delivery system (CLADS, Anesthesia Concerto, Yifeihuatong Corp, Beijing, China); Intravenous injection of 0.6 mg/kg rocuronium, followed by infusion speed at 0.45 mg/kg/h, and stopped 1 hour before the end of the operation.

**Figure 1:**
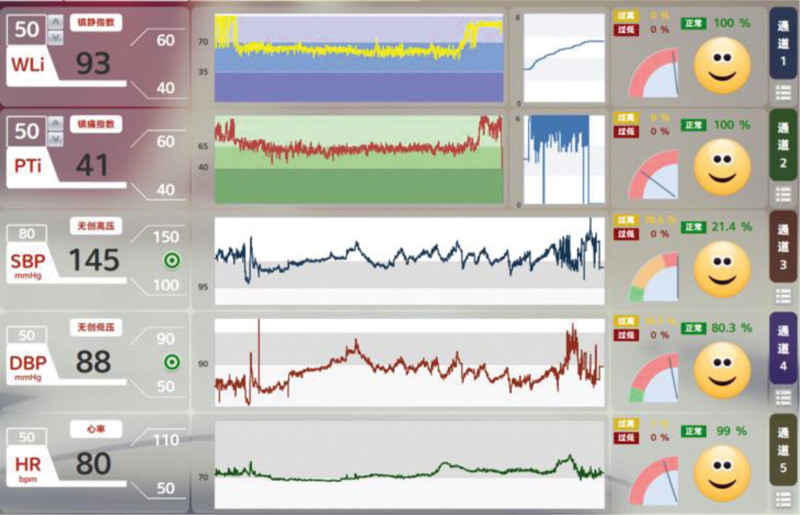
. Trend graphs of WLi, PTi, SBP, DBP and HR. WLi = wavelete index, PTi = pain threshold index, SBP = systolic blood pressure, DBP = diastolic blood pressure, HR = heart rate.

**Figure 2:**
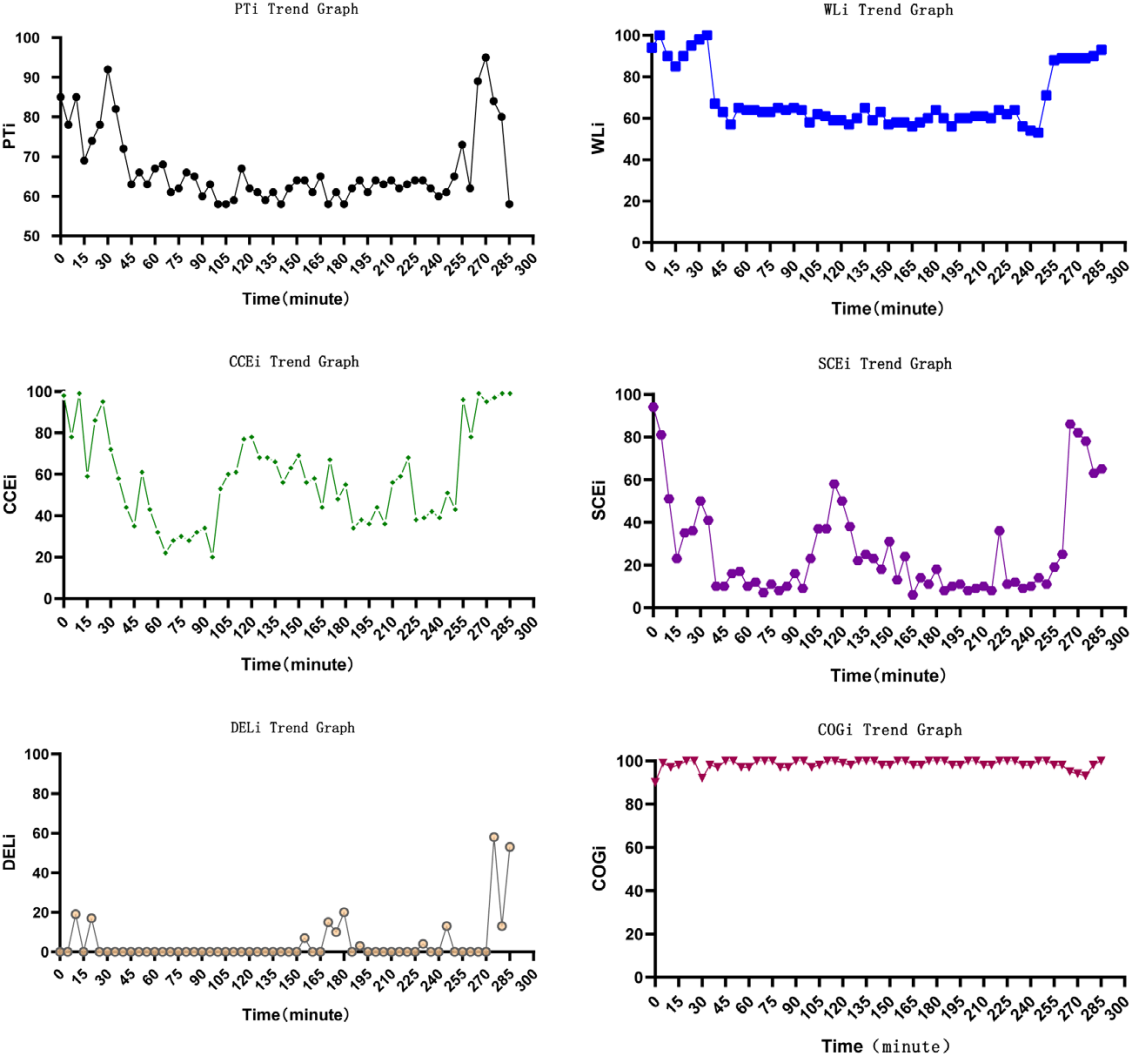
. Trend graph of WLi, PTi, CCEi, SCEi, DELi and COGi. WLi = wavelete index, PTi = pain threshold index, CCEi = cerebral cortical excitability index, SCEi = subcortical excitability index, DELi = delirium index, COGi = cognitive index.

During the anesthesia, there no hypotension occurred, and norepinephrine was not administered. After the patient was converted to open surgery due to intestinal adhesions, the blood pressure (BP) was significantly elevated, and urapidil and esmolol were intermittently injected to reduce BP. After the surgery, the patient received flumazenil (0.5 mg). The duration of the anesthesia was 220 minutes. The recovery procedure from the end of anesthesia to a WLi > 80 was 20 minutes, and the extubation time was 25 minutes. The patient was discharged 7 days after surgery. Figure 1 shows that during surgery, the depth of sedation (wavelete index [WLi], range from 35 to 70) and depth of analgesia (PTi, from 40 to 60) were achieved. When the procedure was switched to an open operation, the patient’s PTi, cerebral cortical excitability index (CCEi) and subcortical excitability index (SCEi) increased and the WLi values were stable (Fig. 2). Intraoperative SVV < 13% was maintained by peripheral intravenous fluid infusion.

## 3. Discussion

The patient underwent radical right hemicolectomy with remimazolam and remifentanil without hypotension. Norepinephrine was not used during the period of anesthesia, whereas urapidil and esmolol were applied to control hypertension after the operation was converted to open.

Patients with HOCM receiving GA should proceed with caution to prevent hypotension and tachycardia which can lead to LVOT obstruction and myocardial ischemia.^[3]^ Similar to other intravenous anesthetics, remimazolam induces amnesia, sedation, hypnosis and immobility.^[4]^ Dong et al^[5]^ reported that the incidence of hypotension in patients undergoing endoscopic retrograde cholangio-pancreatography was lower among those sedated with remimazolam than with propofol. Kim et al^[6]^ reported that remimazolam can be safely used for the induction and maintenance of GA in patients with severe aortic stenosis when performing transcatheter aortic valve implantation. Induction of anesthesia with remifentanil is beneficial for mitigating potentially hazardous haemodynamic responses from stressful stimuli pre-intubation, limiting systemic hypotension post induction.^[7]^ WLi reduced from 98 to 58 and PTi decreased from 98 to 65 with 0.3 mg/kg remimazolam and automatic infusion remifentanil. The systolic BP of our patient decreased from 160 to 140 mm Hg (12.5%) after tracheal intubation. Such hemodynamic stability of remimazolam and remifentanil is expected to play a key role in preventing GA induction hypotension. The BP of patient with gradually increased and significantly increased after switching to open surgery. The patient received ulapidil, esmolol, and more remifentanil with CLADS to reduce the BP. Open surgery is associated with greater trauma, stress response, and sympathetic stimulation than laparoscopic surgery. This may be because remimazolam can act on adrenergic receptors, inhibit norepinephrine release, and reduce the catecholamine level as well as sympathetic nerve excitability, however remimazolam can accelerate atrioventricular conduction and enhance myocardial contractility.^[8]^ The use of esmolol is ideal for suppressing sympathetic stimulation, thereby decreasing the LVOT gradient and improving the cardiac output.^[9]^

The DoA was monitored using CFSi based on electroencephalogram in this patient. Several studies have reported that the bispectral index, patient state index, and narcotrend indices are inappropriate for measuring the sedative effects of remimazolam.^[10]^ After the surgery was switched to open surgery, there were no significant changes in WLi. However, the CCEi and SCEi values of the patient showed a sharp increase, and there was no awareness or recall in the patient. Further research is required on remimazolam and DoA monitoring is warranted. No postoperative complications such as remimazolam-related delirium or cognitive dysfunction were observed in this study.

## 4. Conclusion

In this patient with HOCM, remimazolam and remifentanil provided adequate anesthesia for induction and maintenance to complete right hemicolectomy. Although hypotension and LVOT obstruction did not occur, hypertension did occur. Further clinical studies of remimazolam are desirable to increase its clinical reliability of remimazolam for GA.

## Author contributions

Investigation: Yongchang Shi.

Data curation: Chengchao Zuo, Fengjiao Zhang.

Writing—original draft: Yiwen Zhang.

Software: Chao Zhou.

Supervision: Xuelian Zhao.

Writing—review & editing: Xuelian Zhao.
